# The Role of Caregiver’s Feeding Pattern in the Association between Parents’ and Children’s Healthy Eating Behavior: Study in Taichung, Taiwan

**DOI:** 10.3390/children8050369

**Published:** 2021-05-08

**Authors:** Hung-En Liao, Yueen-Mei Deng

**Affiliations:** Department of Healthcare Management, Asia University, No.500, Lioufeng Rd., Taichung 41354, Taiwan; heliao@asia.edu.tw

**Keywords:** feeding behavior, food behavior, dietary intake, high-fat high sugar (HFHS), early childhood

## Abstract

While parenting style has been linked with parent feeding behavior (FB), little is known about the role FB plays in the relationship between parents’ eating behavior (PEB) and children’s eating behavior (CEB). Based on social learning theory, we hypothesized that children learn to develop healthy CEB by obeying and imitating parents’ healthy eating and feeding behaviors, and that FB is the mediator between PEB and CEB. In total, 257 survey responses from parents of children up to 5 years old were included in the study. Results indicated that CEB did not differ by children’s age, gender, or birth order; for parents, older age and lower educational levels were associated with less healthy unhealthier FB and PEB. Healthy PEB and FB explained 46.8% and 21.7% of the variance in healthy CEB, respectively. The model confirmed that FB reduced the coefficient of healthy PEB from 0.563 to 0.468 and increased the variance explained from 35.0% to 38.5%. FB was a mediator for PEB and CEB. Discussion covers the complexity of ideal parenting styles and child feeding and their associated effects on CEB in varied environments, including different cultures. We concluded that PEB was the main predictor of CEB, and healthy feeding acted as a mediator.

## 1. Introduction

Children’s first five years are a critical period of rapid mental and physical growth, core to which is their nutrition. The World Health Organization (WHO) has proclaimed that a healthy diet and healthy eating help prevent all forms of malnutrition as well as non-communicable diseases (NCDs) such as diabetes, stroke, cancer, and cardiovascular diseases [1]. Extensive studies have also proven that a healthy diet has positive associations with the promotion of both physical (such as preventing cardiovascular diseases [2] and metabolic disorder [3]) and mental health [4], and accordingly reduces risks for chronic diseases, mental disorders, and mortality risk [5] in children’s future lives. At least in the short-term, a healthy diet is closely associated with children’s cognitive and executive functioning [6] and school performance [7]. Food eating behavior is a simple method for nutrient intake, and healthy eating is a complex human behavior for children to learn [8]. The main source of such learning is the parents or caregivers. 

The literature confirms a significant connection between the feeding behavior of parents and the eating habits of children [9,10,11,12,13,14,15,16]. In contrast to western cultures, parenting and feeding practices in eastern cultures, specifically the Chinese culture in Taiwan, differ regarding parents’ expectations of their children. In Chinese communities, parents are stricter, more authoritative, and more likely to request that children follow parents’ orders and instructions without question. Unlike parents from western cultures, Chinese parents appreciate children for accomplishments that meet the parent’s expectations, not what the child loves to do. Parenting styles in a Chinese family consider the child’s future academic performance and job achievement rather than love for the child [17]. Parenting styles have been used in the literature [18,19] to study the essence, quality, and outcomes of the connection between parents and children, but it has been less conclusive in Chinese families, even those that take cultural context into consideration [20]. 

Irrespective of the strength of the correlations among parenting style, parent feeding strategies, and parent and child eating behavior, a major concern is the health of these behaviors and how they are connected in non-western communities. 

Healthy diets can ensure that humans get adequate and proper nutrition for growth and survival; unhealthy diets will cause excessive load on the body, impair physical activity, and impair physical and mental functions [21]. Another concern is the roles that parents’ healthy feeding and parents’ healthy eating behaviors play in such an association. Drawing on social learning theory (SLT) [22], we proposed that children’s healthy eating behavior is affected by parents’ eating behavior through parents’ healthy feeding practices.

Eating behaviors are formed at or before the preschool years, but numerous factors may contribute to their development. These include genetic predispositions, food preferences, and eating practices, as well as social and environmental factors, including parental factors [23,24,25,26,27,28,29,30,31,32]. Cultivating healthy eating habits from childhood not only significantly contributes to the personal growth and future health of adults, but also lays an important foundation for overall social health.

As one of the major determinants of children’s eating behavior [32], parental healthy eating behavior has a distinct role in explaining BMI reduction [8,33,34,35,36]. As SLT [22] suggests, parents ’ eating habits can act as a model to be imitated. In SLT, the child may learn to behave in a desired way through the effects of stimuli (such as the parent’s control of food intake to gain desired rewards) and environmental factors (such as imitating the behavior of human subjects) [36,37]. Literature has shown that parents’ healthy diets contribute significantly to fostering children’s healthy eating behaviors [38] since children tend to develop their eating behavior through observing and imitating people in their environment [39]. Core to children’s learning of healthy eating is the communication between children and their parents about food at early ages [40,41,42,43], in particular before the age of five. Communications between parents and children on healthy eating may be performed through both verbal and non-verbal (e.g., body language) communication [44,45]. Non-verbal effects may come from parents’ actual healthy eating behavior [45,46].

Based on SLT, we may conclude that children’s healthy eating behavior results from their imitation of parents’ healthy eating behavior, with hypothesis 1 (H1) stating that the parent’s healthy eating behavior has positive effects on their children’s healthy eating behavior.

Parents with unhealthy eating behavior are less demanding on their children’s healthy eating [34]; this implies that, healthy or not, parents’ eating behavior will affect their level of control over their children’s food intake.

Parents’ child feeding behavior, just like parents’ eating behavior, is an integrated part of the parent’s behavioral model. Parents develop their own eating behavior through a socialization process that begins early in their childhood and then becomes a foundation for their child-feeding practices. In addition, as suggested by SLT, parents’ healthy eating is affected not only by their caregivers but also by their environment. Evidence for this is seen in how healthy eating behavior is closely related to higher education [47]. Higher education means broader access to health information and healthy eating behavior. Healthy child feeding is another example from which parents can observe and learn from their peers in their personal network, as SLT advocates. Commonly, husbands in Chinese families hold at least the same level of education as their wives. A mother with higher education means healthier eating and child feeding in that family. By appreciating the benefits of healthy eating behavior and being affected by the positive models around them, parents will be more willing to practice healthy feeding. 

The current research thus proposes hypothesis 2 (H2): parents’ healthy eating has positive effects on parents’ healthy feeding behavior.

Looking further into the concept of parent’s feeding styles, studies have identified three major aspects that affect this particular issue: pressuring (e.g., pressure to eat), restriction (e.g., restriction on high fat and high sugar, HFHS, food or snack), and instrumental feeding (e.g., food as a reward), along with other emerging aspects such as parental imposition regarding mealtimes and places [48]. The consequences of these parenting activities received vast attention, particularly regarding children’s physical and mental health [9,10,15,25,35,48,49]. Noteworthy is the fact that pressure to eat can be detrimental to the child’s food intake [50] and can decrease the child’s food preference [51].

Literature has suggested that parents’ excessive control of food, such as in authoritarian feeding with high demand and low responsivity, as well as excessive permissiveness, such as negligent and indulgent feeding with low demand and low responsivity, negatively affect their children’s healthy eating behavior [13,17,18,50,51,52]. The parent’s child-feeding style is part of the parent’s eating experience, particularly for those who were grown with bad food intake behavior. This accordingly contributes to unhealthy eating behavior that developed in the parent’s childhood [31].

On the other hand, the literature also suggests that feeding style is one of the two major determinants of children’s eating behavior [32], and perceived parental healthy eating behavior plays a distinct role in explaining BMI reduction among overweight adolescents [46]. This means that children may establish their eating behavior through their parents’ feeding style, which is mostly delivered through explicit instructions, as well as the parent’s eating behavior, which provides a model that children may imitate, as SLT suggested.

For preschool children in Chinese communities, the nature and magnitude of the association among parents’ eating behavior, parental feeding behavior (mostly through verbal instruction), and children’s eating behavior have not been established. Based on the previous review, we hypothesized that parents’ habitual eating behavior affects their child-feeding and their children’s eating behaviors. Further, the parents’ healthy eating habits are part of their daily behaviors. Similar to their habitual eating, child feeding is one of their numerous daily and habitual behaviors, and parents’ habits will influence their children’s healthy eating behavior through healthy child feeding behavior. Parents’ healthy feeding is generally formed after their healthy eating behavior develops, and thus we propose hypothesis 3 (H3): healthy child feeding behavior mediates the link between parent and child healthy dieting behavior.

## 2. Materials and Methods

### 2.1. Materials

Research material is the data gathered from parents who have preschool children enrolled in kindergartens in the Taichung Metropolitan area in Taiwan. Participants responded to posters inviting them to volunteer for a research study. Participants were asked to complete a paper questionnaire during parent-teacher conferences.

**Figure 1:**
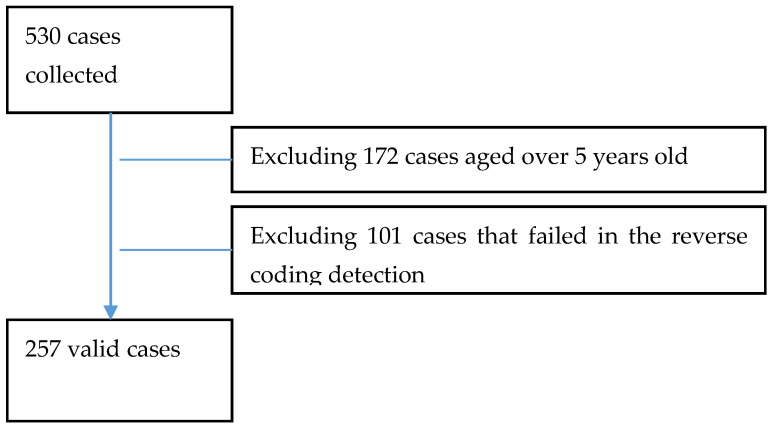
Valid subjects entered in the study.

The literature suggests that children’s nutrition requires special attention in a pre-school setting [52] to avoid overeating and weight gain [5,35], especially for children between age two and five. Out of 530 participants, 358 subjects had children aged no more than five years old. In the data cleaning stage, we further exclude 101 responses that were identified as invalid through a comparison of reversed questions. Overall, 257 cases remained valid for further analyses, as shown in Figure 1. 

### 2.2. Measurements

A structural questionnaire was developed referencing the major literature regarding parent’s feeding and children’s eating behaviors in Supplementary Materials. Items included in this research instrument referred to an array of research, such as the Child Feeding Questionnaire (CFQ) [53], and the Caregiver’s Feeding Styles Questionnaire [54] for parents’ healthy feeding behavior. Items for children’s healthy eating behavior are drawn from The Child Eating Behavior Questionnaire [49,55]. These instruments regarding parenting styles and child feeding behavior provided valuable references to develop the scale specifically for the current research [13].

In our instrument, eating behavior included: sufficient vegetable and fruit intake, snacking, chewing slowly, TV-watching while eating, taking regular meals, hygienic eating behaviors (e.g., hand washing and tooth brushing, picky eating, and rest after meals), staying with their food, and staying quiet while eating, among others. Parents’ feeding behavior included: preparing meals with varied cooking methods, encouraging children to try new kinds of food, setting a regular time and place for eating, prohibiting HFHS or energy-dense foods, and requesting children eat all of the kinds of food on their plate, among others.

Based on social learning theory [22], this study proposed that children learn from observing and imitating their caregiver’s eating behavior during their socialization process. The study uses some explicit items from the children’s eating behavior measurement as an assessment of the parent’s eating behavior, such as sufficient vegetable and fruit intake, snacking, TV-watching while eating, and an additional item about updating dieting information specifically for the parents.

The questionnaire and research framework were sent to five external experts to assess validity. In the second round, these experts evaluated each item as “healthy” “not healthy” or “neutral” behavior in their judgment. Experts included one nutritionist from a medical center, one pharmacist from a large-scale community pharmacy, two professors who teach food nutrition courses in the Hospitality Department and the Food Science and Technology Department, and one senior teacher from a local kindergarten. 

A 5-point scale (1 to 5) was used to determine the respondent’s psychometric properties on each variable. “1” represents “0% of occurrence” and “5” represents “75–100% of occurrence” for the measurements of caregiver and child healthy eating behavior; whereas “1” represents “highly disagree” and “5” represents “highly agree” for parent feeding policies. Unhealthy behaviors were designed as reverse-scored questions. Average scores and standard deviations were then calculated to indicate the respondent’s level of healthy behavior on each construct. The higher the score, the healthier the respondent’s behavior. 

Reliability of each construct was examined for internal consistency, with a Cronbach’s α = 0.714 for the parents’ healthy eating behavior, 0.876 for the children’s healthy eating behavior, and 0.844 for the parents’ healthy feeding behavior. 

The study, including the research plan and measurement instrument, was approved by the Institutional Review Board (IRB) of Jen-Ai Hospital (Taichung, Taiwan) with a No. 109-38 before the study was conducted. Participants were informed that they are entitled to terminate their participation during the survey, and signed a written consent before a survey was conducted. 

### 2.3. Analyses

This study used SPSS 20.0 (Armonk, NY: IBM Corp. Sourced from TriStar, Kaohsiung City, Taiwan) to analyze the data. Other than analyzing the data distribution and testing the reliability of each variable, we adopted independent *t*-tests and one-way ANOVAs followed by Scheffe’s method as a post hoc test for analyses with a significant *F*-statistic to determine which pairs of means are significant. We also used hierarchical regression analysis to assess the effects of parents’ healthy eating behavior on parents’ healthy feeding patterns and children’s healthy eating behavior, and then to examine the mediating effects of healthy feeding patterns on the association between parent and child healthy eating behaviors.

## 3. Results

### 3.1. Demographic Distribution of Subjects

There were 257 valid responses included in the current study. Children were 52.92% male, with 14.01%, 37.74%, and 48.25% of children aged under 3, 3–4, and 4–5 years of age, respectively. 42.80% were the second or younger children in the family, shown in Table 1. Primary caregivers were mostly mothers (84.82%), with 66.54% of them aged between 31 and 40, and 94.55% at least high school educated.

### 3.2. Analyses of Variables

Children’s healthy eating behavior ranged from 3.40 ± 0.42 to 3.58 ± 0.40 with an average of 3.51 ± 0.37 on a 5-point scale across different categories. Scores for parents’ eating behavior ranged from 3.31 ± 0.38 to 3.63 ± 0.42 across categories of caregivers, age, and education with an average of 3.54 ± 0.46. Parents’ feeding practices ranged from 3.60 ± 0.43 to 4.00 ± 0.41 with an overall average of 3.83 ± 0.45.

Regarding children’s gender, healthy eating behavior was at an equal level (*p* = 0.240, > 0.05) for boys (M = 3.54, SD = 0.37) and girls (M = 3.48, SD = 0.37). Second and younger children had significantly higher scores (*p* = 0.022) than their older brothers or sisters (M = 3.58, SD = 0.40), and the children aged between 4 and 5 years old had healthier eating behavior (M = 3.51, SD = 0.37) on average, but this was not statistically significant (*p* = 0.064). 

Children’s mothers had significantly healthier eating (3.58 ± 0.46) and feeding behaviors (3.31 ± 0.38) compared to the other caregivers, with *p* < 0.001 and *p* = 0.032, respectively. We further explored whether healthy eating and feeding behaviors were different in terms of the caregiver’s age and education levels. As Table 2 indicates, caregivers who were aged over 46 were significantly less healthy in child-feeding (3.61 ± 0.39) than younger-aged caregivers, with *p* = 0.008. For the parents’ healthy eating behavior, though the caregivers aged over 46 years again featured the lowest scores in healthy eating behavior, no significant differences were found among age groups (*p* = 0.082). 

Caregivers’ education levels were associated with different average scores in healthy eating and feeding behaviors as well. Highly educated parents had the highest scores in healthy eating behavior (3.62 ± 0.45), significantly higher than those who received high school education (3.37 ± 0.51) with *p* = 0.003.

### 3.3. Association between Parents’ and Children’s Healthy Eating Behavior

As we hypothesized in H1 and H2, results showed that the parent’s healthy eating behavior had positive effects on their children’s healthy eating behavior (*β =* 0.563, *p* < 0.001, *R*^2^ = 0.350), as M4 in Table 3 shows, as well as on their children’s feeding behavior (*β* = 0.435, *p* < 0.001, *R*^2^ = 0.270), as M2 in Table 3 shows.

Combining the effects of parents’ healthy eating and feeding behaviors, the effect of PEB reduced from 0.563 to 0.468, which explained more of the variance in CEB, increasing from 35.0% to 38.5%, of which PEB remained as a main predictor (*β* = 0.468, *p* < 0.001), followed by FB (*β* = 0.217, *p* < 0.001) as a mediator. This means that parents’ healthy eating behavior significantly affected their children’s healthy eating behavior partially by way of healthy feeding patterns. Results indicated that healthy feeding partially mediated the link between parent–child healthy eating behavior [56], as M5 in Table 3, supporting our hypothesis.

**Figure 2:**
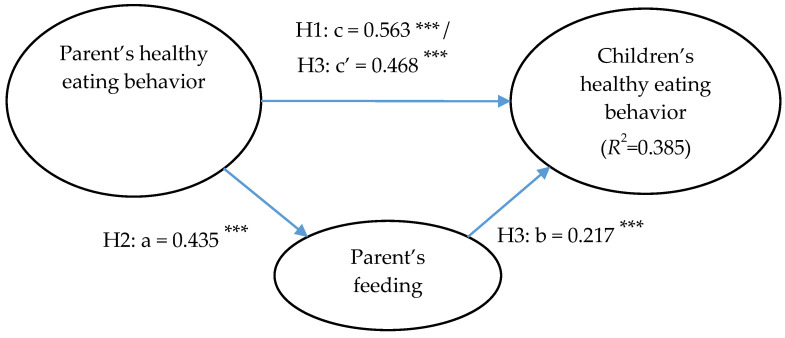
Parent’s healthy feeding as a mediator of parents’ and children’s healthy eating behaviors (*** *p* < 0.001).

The overall associations between parents’ healthy eating and feeding behavior and children’s healthy eating behavior showed that children’s healthy eating behavior is affected by both following their parents’ feeding style and imitating the parents’ healthy eating styles. The parents’ feeding style was affected by their own eating behavior and acted as a mediator in the association between PEB and CEB, as shown in Figure 2.

## 4. Discussion

A healthy diet and healthy eating help prevent a wide range of NCDs [1]. It is therefore important to foster children’s healthy eating habits as early as possible. As part of health education, a child’s parents and caregivers are essential in executing this mission. 

In this study, children’s healthy eating behavior was low across all categories of gender, birth order, and age. Healthy eating behavior has multiple benefits for children’s mental and biological growth, as well as their future development. The low overall est scores on children’s eating behavior in this study revealed that this behavior needs to be improved. The items that received the lowest scores, such as slow eating, TV-watching during eating, and snacking between meals, are the most noticeable ones. TV-watching and smartphone addiction are strongly associated with obesity and other health problems such as future metabolic syndrome [57,58]. Slow eating usually accompanies unhealthy eating behaviors such as picky eating, with negative consequences for children’s health. Parents should remain alert for these unhealthy behaviors, and consult with health professionals (e.g., pediatricians) for possible eating disorders such as anorexia nervosa (AN). Snacking is not always a problem or a threat to children’s health. However, while snacking has some health benefits such as appetite control and weight management, it is still a major contributor to weight problems [59]. Major concerns about snacking center on their HFHS ingredients as well as how the quantity and timing of snacking reduces appetite during regular meals, resulting in either excessive or insufficient nutrition for children’s growth [60]. 

Healthy child-feeding is important for children’s growth, as it provides guidance for children to follow and develop healthy eating habits around. Since children may learn by imitating the eating behavior they see in others, an ideal care plan should cover healthy feeding behavior as well as imitable healthy eating behavior across the entire eating environment, including with family, in school or kindergarten, and/or in restaurants. Given that mothers remain the primary caregivers and significantly influence their children’s healthy eating habits, accessible resources should be made available to them, including healthy eating and feeding information (including efficient and diversified cooking methods, nutrition knowledge, child-feeding practice, and other strategies) and conferences on healthy child-feeding. Mothers were the best performers of healthy feeding and eating behaviors in this study, significantly superior to non-mothers such as fathers and grandparents. Although the current scores for mother’s eating and feeding behaviors are not healthy at a satisfactory level, it is encouraging that mothers have higher scores and are in a better position to improve their healthy eating and child-feeding practices.

The current research indicated that healthy eating and feeding behaviors are varied across different age groups and parent education levels. Caregiver’s age and education accounted for differences in many behaviors, including eating and child-feeding [47,61,62,63,64,65,66]. Caregivers in the oldest age group are less healthy in child-feeding. Since the average age of mothers who had their first child between 2014 and 2016 were 30.51 to 30.74 years old, and only 0.11–0.17% were aged older than 45 years old [67], older caregivers are likely the child’s senior relatives, e.g., grandparents. Grandparents are generally less attentive and responsive to children’s psychoemotional needs and exhibit different custodial attitudes and practices in child care [68,69]. Regarding education levels, grandparents are less educated than the younger generation in Taiwan [67], and thus possess less health literacy and accordingly more unhealthy eating behaviors [70]. Grandparents can be reliable caregivers with multiple benefits, particularly when mothers have full-time jobs [69,71]. The crucial point is consistent child-care styles across all caregivers, without conflicts between grandparents and parents, to avoid potentially unhealthy feeding practices [69] and bad examples. An effective and accessible support system for custodial grandparents that can act as a buffer against negative consequences will be particularly important for highly industrial eastern countries [69]. 

The literature has generally reached a consensus on the relationships among parental feeding patterns, parental eating habits, and children’s eating habits, but it has not specifically looked at the concept of healthy eating and feeding. This study demonstrates that parents with healthy eating habits will be more capable of designing and implementing a healthy dieting plan for their children. Therefore, the parent’s healthy eating habits are the antecedents of the parent’s healthy feeding patterns. Past studies have successfully shown the close relationship between parent and child eating behavior, yet some argue that the parent’s feeding style is the determinant of their children’s healthy eating. Drawing from social learning theory, we proposed and confirmed with empirical data that children learn to establish healthy eating behavior through habitual food intake provided by parents in the infant period, and later through observing and imitating the parent’s eating behavior. Parents’ eating process is critical in the process of building healthy behavior in children, as parents’ eating drives their healthy feeding behavior and provides a benchmark or model for the children to imitate.

Conforming to previous research, the current study confirms that children’s healthy eating behavior is affected by parents’ eating behavior [30,48], which is partially mediated by parents’ feeding behavior. A child eats what the parents feed them in the infant stage. Later, at preschool age, i.e., 3–6 years old, they develop their dieting habits by following the parent’s feeding instruction and imitating the parent’s eating behavior [22,44,45]. When parents eat healthily, they will likely feed their children in the same manner, and accordingly, the children eat healthily as well.

Snacking between meals typically means eating without hunger and can be a major source of HFHS intake, which needs to be well-controlled. Like TV-watching while eating, providing snacks may act as a convenient incentive to encourage children’s healthy eating, but trade-offs of this kind tend to foster unhealthy dieting habits in children’s later life [59,60].

Other than healthy feeding, the environment is important to help children foster healthy eating habits [72]. Exposure to an environment that features HFHS meals along with eating patterns within such an environment are certainly a bad guide for unhealthy eating behavior, as social learning theory suggests. Eating out of the home risks placing children in unhealthy eating environments, as it tends to provide easy access to HFHS foods and examples of unhealthy behavior to imitate. Children eating at home with their parents is ideal to foster healthy eating behavior, however, this is becoming harder than ever in some places, such as metropolitan areas in Taiwan and Asia. 

The national culture may have an impact on selecting ideal parenting and feeding styles. Although the causal pathways of these differences remain uncertain, cultural differences must certainly have an impact on parenting styles and child feeding patterns. National culture dimensions [73] have been associated with body mass index (BMI) [74]. Philosophical rationales behind such cultural differences may affect the parent’s choice of parenting and feeding styles, and thus the ideal parenting style may vary from one culture to another [75]. An ideal project for the development of children’s healthy eating behavior should consider the culture-specific aspects of parent’s feeding style as well as how can that be achieved [76]. Though diet habits and ideal feeding style may vary from culture to culture, parents play a pivotal role in how children learn to build healthy dietary practices in their early life [1,76]. In other words, healthy or not, parents’ eating and feeding can be a larger contributor to children’s healthy eating behavior under a specific cultural context. 

Consistent with previous work [32,49], parents’ feeding policies and eating behavior in this study were found to affect children’s healthy eating behavior. Based on social learning theory, the current research extends our understanding of children’s healthy eating behavior, showing that it is a combination of children’s imitation of their parent’s healthy eating behavior and their parent’s healthy feeding practices. On the other hand, the parent’s healthy feeding is a combination of their healthy eating experience as well as mutual learning from other families in Chinese communities. This finding suggests that parents’ communication with their children about healthy eating behaviors should be verbally and non-verbally consistent; in other words, an example is better than precepts. 

## 5. Conclusions

A healthy diet is vital to human growth and development in terms of physical and mental health as well as academic and career performance. Children develop healthy diet patterns in their early life as a part of their socialization, learning from their parents’ feeding instructions and diet practices. As social learning theory suggests, the current study confirmed that children not only follow parents’ instructions, but also imitate parents’ behavior in dieting. The current study revealed that parents’ child-feeding behavior mediates the association between parents’ healthy dieting and children’s healthy eating behavior. We suggested that a child-feeding guide could be designed to remind parents or caregivers to update their nutrition knowledge, create a healthy eating atmosphere, restrict the accessibility of unhealthy foods, and practice a healthy diet pattern.

### Limitations of the Study

The first limitation of the study is some of the possible factors that may contribute to the children’s healthy or unhealthy eating behavior were not included in the study, such as nutrition, physical exercise, parent’s nutrition literacy, and family leisure activities, among others. 

The second limitation may be that the subjects included in the current research were limited to parents who have children enrolled in one of the selected kindergartens in the Taichung metropolitan area. Though the city is the second-largest in Taiwan and well-diversified in the population composition in terms of age, gender, and other demographic factors, the sampling process was not random. Compared to a rural area, the residents in urban generally benefit from having higher incomes, better occupations, and superior living facilities. Results of the current research may not be generalizable to other populations.

Regarding the use of the term “affect “ and “effects”, we are aware of Reichenbach’s principle [77] on the direction of time, and therefore we do not claim to determine causality of independent and dependent variables.

## Figures and Tables

**Table 1:** Demographic distributions.

Factors	Category	*n*	%
Preschool children			
Gender	Male	136	52.92
	Female	121	47.08
Age (Child)	<3	36	14.01
	3–4	97	37.74
	4–5	124	48.25
Order of child	Only child	75	29.18
	Oldest	72	28.02
	Second & Younger	110	42.80
Parent or major caregiver			
Relationship	Mothers	218	84.82
	Others	39	15.18
Age (Parent)	<30	34	13.23
	31–35	81	31.52
	36–40	90	35.02
	41–45	25	9.73
	>46	27	10.51
Education	<High school	14	5.45
	High school	55	21.40
	Vocational college	28	10.89
	Bachelor’s & up	160	62.26

*n* = 257.

**Table 2:** Mean, standard deviation, *t*-test, and ANOVA of variables.

Variables	Group	*n*	M	SD	t/F	*p*	Post hoc ^b^
**Children**	*Healthy Eating*						
Gender	Male	136	3.54	0.37	1.18	0.240	-
	Female	121	3.48	0.37			
Birth order	Only child	75	3.50	0.30	3.90	0.022	Only child > Oldest
	Oldest	72	3.42	0.37			
	2nd+ ^c^	110	3.58	0.40			
Age	<3	36	3.40	0.42	2.77	0.064	-
	3–4	97	3.56	0.35			
	4–5	124	3.51	0.37			
	Overall	257	3.51	0.37			
**Parents**	*Caregivers*						
Healthy Eating	Mothers	218	3.58	0.46	3.86	<0.001	-
	Non-mothers	39	3.31	0.38			
Health Feeding	Mothers	218	3.86	0.46	2.19	0.032	-
	Non-mothers	39	3.70	0.39			
**Parents**	*Age*						
Healthy Eating	<30	34	3.53	0.52	2.10	0.082	-
	31–35	81	3.50	0.43			
	36–40	90	3.61	0.47			
	41–45	25	3.63	0.42			
	>46	27	3.35	0.40			
Healthy Feeding	<30	34	3.74	0.51	3.55	0.008	(>46) < (31–35), (36–40), (41–45)
	31–35	81	3.81	0.49			
	36–40	90	3.91	0.40			
	41–45	25	4.00	0.41			
	>46	27	3.61	0.39			
**Parents**	*Education*						
Healthy Eating	<HS ^a^	14	3.43	0.32	4.88	0.003	Bachelor > HS
	HS ^a^	55	3.37	0.51			
	VET. Coll ^a^	28	3.47	0.34			
	Bachelor + ^d^	160	3.62	0.45			
	Overall	257	3.54	0.46			
Healthy Feeding	<HS ^a^	14	3.60	0.43	1.35	0.260	-
	HS ^a^	55	3.85	0.51			
	VET. Coll ^a^	28	3.81	0.37			
	Bachelor + ^d^	160	3.85	0.45			
	Overall	257	3.83	0.45			

^a^ HS, High school; VET. coll, Vocational Education and Training college. ^b^ Posthoc: Scheffe’s method is applied for pairs with a significant *F*-statistic. ^c^ 2nd+, the second child or younger. ^d^ Bachelor+ a bachelor degree and above.

**Table 3:** Mediating effects of parent’s healthy feeding.

Variables	Healthy FB		Healthy CEB		
	M1	M2	M3	M4	M5
C. Gender	−0.005	0.041	−0.111	−0.051	−0.060
C. Age	0.291 ***	0.203 ***	0.084	−0.030	−0.074
C. Birth order	0.047	0.039	0.071	0.060	0.051
P. Caregiver	−0.157 *	−0.058	−0.221 **	−0.092	−0.079
P. Age	0.047	0.004	0.090	0.034	0.033
P. Education	0.041	−0.042	0.103	-0.005	0.004
Independent Variable					
Healthy PEB		0.435 ***		0.563 ***	0.468 ***
Mediator					
Healthy FB					0.217 ***
*R^2^*	0.104	0.270 ***	0.074 *	0.350 ***	0.385 ***
*Adj. R^2^*	0.083	0.249 ***	0.052	0.332 ***	0.365 ***
△*R*^2^		0.166		0.277	0.034
*F*	4.86	13.155	3.321	105.989	13.884

*n* = 257; C, children; P, parent; FB, parent’s feeding behavior; PEB, parent’s eating behavior, CEB, children’s eating behavior; *R*^2^, *R*-squared; *Adj. R*^2^, adjusted *R*^2^; △*R*^2^, *R*^2^ change; *F*, *F* statistic value; * *p* < 0.05; ** *p* < 0.01; *** *p* < 0.001.

## Data Availability

The data presented in this study are available on request from the corresponding author. The data are not publicly available due to the confidential requirements required by the university department. Data can be available on request subject to the department approval.

## Supplementary Materials

The following are available online at https://www.mdpi.com/article/10.3390/children8050369/s1.
