# Supplementary material for: The Role of Caregiver’s Feeding Pattern in the Association between Parents’ and Children’s Healthy Eating Behavior: Study in Taichung, Taiwan
Source: Children (Basel). 2021 May 8;8(5):369. doi: 10.3390/children8050369 (PMC8151811; doi:10.3390/children8050369)
Supplement: Supplementary file 1 [file children-08-00369-s001.zip › children-1177312-supplementary.pdf]

A1. Children's current height: cm (please fill in)
A2. Children's current weight: kg (please fill in)
A3. The gender of the child: □(1) male □(2) female
A4. Age of children: □(1)under 3 years old □(2)□over 3 years old to under 4 years old (3) □over 4 years old to under 5 years old (4)
5 years old to less than 6 years old □ (5) 6 years old (inclusive) and above
A5. Infant’ s birth order: □(1) Only child □(2) Boss □(3) Second child □(4) Third child
□(5) Fourth (inclusive) and above
A6. Living area: □(1) Taichung City (District) □(2) Nantou County (City) □(3) Changhua County (City)

A7. The main caregiver of the infant's diet: (single choice)
□(1) mother □(2) father □(3) grandparents
□(4) Nanny □(5) Other relatives

A8. The age of the main caregiver for the infant's diet:
□(1) Under 25 years old (inclusive) □(2) 26-30 years old □(3) 31-35 years old □(4) 36-40 years old
□(5) 41-45 years old □(6) 46-50 years old □(7) 51 years old (inclusive)
A9. Educational level of the main caregivers of young children's diet:
□(1) Elementary school □(2) Junior high school and below □(3) Junior high school
□(4) High school (vocational) □(5) Junior college □(6) University and graduate school

Items in the questionnaire for healthy eating and feeding practices

A. Parent's healthy eating behavior

1. Never: 0% of occurrence<br>2. Rarely: up to 25% of occurrence<br>3. Sometimes: about 25~50% of occurrence<br>4. Often: 51~75% of occurrence<br>5. Always: 75~100% of occurrence	Mean	S. D.
B1. I watch TV while eating.	3.14	0.93
B2. I chew slowly when I eat.	3.71	0.99
B3. I pick up the food on the table and eat it.	2.94	0.90
B4. I collect and receive updated information about diet and health continuously.	3.61	0.93
B5. I take snacks (such as chocolate, biscuits, cakes, etc.).	3.25	0.79
B6. I eat fast food (such as burgers, fryer, pizza, etc.).	3.12	0.68
B7. I drink sugary drinks (such as soft drinks, commercially available juices, milk tea, etc.).	3.21	0.86
B8. I take late night snack	3.09	0.73
B9. I eat at least a fist-sized amount of vegetables every day.	3.84	0.89
B10. I eat at least a fist-sized amount of fruit every day.	3.75	0.98
B11. I am a picky eater.	3.31	0.93
B12. I wash my hands before meals, clean my teeth after meals	3.47	0.75

B1, B3, B5, B6, B7, B8, B11 are reverse questions, and means (and s. d.) figures for these are computed with re-coded values for healthy behaviors.

B. The caregiver's feeding practices

1.“Highly Disagree”,<br>2. “Disagree”,<br>3. “Neutral”,<br>4. “Agree”,<br>5. “Highly Agree”	Mean	S. D.
C1. I think breakfast is important, so I must eat breakfast.	4.50	0.66
C2. Half an hour before the main meal, I do not provide my children snacks	4.31	0.67
C3. I will limit the number and amount of snacks and drinks the child can eat.	4.39	0.61
C4. I will try different cooking methods to make children like food.	4.15	0.69
C5. I will ask the child to help prepare meals to increase his interest in food.	3.96	0.74
C6. When a child tries new food or food that he doesn't like, I will praise him.	4.46	0.60
C7. I let the children have a fixed meal time.	4.22	0.69
C8. I will pay attention to the intake of children's meals.	4.27	0.63
C9. No matter how much or less I eat, I will ask the child to eat every kind of food provided on the table.	3.81	0.93
C10. I think eating is just getting full, and it doesn’t matter whether the nutrition is balance or not.	3.69	1.12
C11. I think picky eating behavior will gradually improve as child grow up.	3.19	1.08
C12. I will feed my children because they eat too slowly.	3.35	1.12
C13. I will take the initiative to buy sugary drinks (such as soft drinks, commercially available juices, milk tea, etc.) for my children.	3.74	1.09
C14. As long as the children are willing to eat, it doesn't matter if they eat and play or watch TV while eating.	3.75	1.00
C15. I think there is no need at all to force a child to eat, he will eat when he is hungry.	3.30	0.99
C16. I think children do not need to eat in a designated place.	3.68	1.09
C17. For three meals, I only prepare the foods that the child likes or prefers.	3.64	1.07
C18. I will let children eat sweets.	3.18	0.93
C19. I will agree to my child's request to go to a fast food restaurant.	3.27	0.97

C10~C19 are reverse questions, and means (and s. d.) figures for these are computed with re-coded values for healthy behaviors.

C. **Children's healthy eating behavior**

1. Never: 0% of occurrence<br>2. Rarely: up to 25% of occurrence<br>3. Sometimes: about 25~50% of occurrence<br>4. Often: 51~75% of occurrence<br>5. Always: 75~100% of occurrence	Mean	S. D.
D1. Child watches TV while eating or play while eating.	3.04	1.05
D2. Child chews slowly when eating.	3.46	0.95
D3. The average time for child to eat exceeds 30 minutes.	2.94	0.95
D4. Child grabs food to eat with their hands.	3.30	0.84
D5. When the child eats, he will sit and not run around until the meal is finished.	3.12	1.03
D6. The child does not talk when there is food in the mouth.	3.02	0.91
D7. Child drinks sugary drinks	3.45	0.94
D8. The child only eats certain types of food.	3.27	0.95
D9. Children eat snacks (such as chocolate, biscuits, candies, etc.).	3.13	0.87
D10. The child eat at least one fist sized amount of vegetables every day.	3.37	0.94
D11. The child will eat at least one fist sized amount of fruit every day.	3.68	0.88
D12. The child eats breakfast every day.	4.54	0.73
D13. The child eats lunch every day.	4.64	0.61
D14. The child eats dinner every day.	4.65	0.64
D15. Child eats more the foods they like.	3.89	0.94
D16. Child eats up all the food in her/his bowl.	3.81	0.95
D17. Child eats the food he dislike as an exchange for other things.	3.83	1.26
D18. Child washes hands before meals.	3.97	1.09
D19. Child cleans teeth after meals.	3.35	1.03
D20. Child takes strenuous exercise within half an hour after the meal.	3.61	1.01
D21. Child helps clean up the tableware after eating.	3.67	1.08

D1, D3, D4, D7~D9, D15, D17, D20 are reverse questions, and means (and s. d.) figures for these are computed with re-coded values for healthy behaviors.

This is the end of the questionnaire, thank you for your participation.
